# Fetal exposure to valproic acid dysregulates the expression of autism-linked genes in the developing cerebellum

**DOI:** 10.1038/s41398-023-02391-9

**Published:** 2023-04-05

**Authors:** Marika Guerra, Vanessa Medici, Robert Weatheritt, Valentina Corvino, Daniela Palacios, Maria Concetta Geloso, Donatella Farini, Claudio Sette

**Affiliations:** 1 Department of Neuroscience, Section of Human Anatomy, Catholic University of the Sacred Hearth, Rome, Italy; 2 GSTeP-Organoids Research Core Facility, Fondazione Policlinico Universitario A. Gemelli, IRCCS, Rome, Italy; 3 Garvan Institute of Medical Research, EMBL Australia, Darlinghurst, NSW Australia; 4 Department of Life Science and Public Health, Section of Biology, Catholic University of the Sacred Hearth, Rome, Italy; 5 Department of Biomedicine and Prevention, University of Rome Tor Vergata, Rome, Italy

**Keywords:** Molecular neuroscience, Human behaviour

## Abstract

Autism spectrum disorder (ASD) includes a set of highly heritable neurodevelopmental syndromes characterized by social and communication impairment, repetitive behaviour, and intellectual disability. Although mutations in multiple genes have been associated to ASD, most patients lack detectable genetic alterations. For this reason, environmental factors are commonly thought to also contribute to ASD aetiology. Transcriptome analyses have revealed that autistic brains possess distinct gene expression signatures, whose elucidation can provide insights about the mechanisms underlying the effects of ASD-causing genetic and environmental factors. Herein, we have identified a coordinated and temporally regulated programme of gene expression in the post-natal development of cerebellum, a brain area whose defects are strongly associated with ASD. Notably, this cerebellar developmental programme is significantly enriched in ASD-linked genes. Clustering analyses highlighted six different patterns of gene expression modulated during cerebellar development, with most of them being enriched in functional processes that are frequently dysregulated in ASD. By using the valproic acid mouse model of ASD, we found that ASD-linked genes are dysregulated in the developing cerebellum of ASD-like mice, a defect that correlates with impaired social behaviour and altered cerebellar cortical morphology. Moreover, changes in transcript levels were reflected in aberrant protein expression, indicating the functional relevance of these alterations. Thus, our work uncovers a complex ASD-related transcriptional programme regulated during cerebellar development and highlight genes whose expression is dysregulated in this brain area of an ASD mouse model.

## Introduction

Autism spectrum disorder (ASD) is a heterogeneous neurodevelopmental disorder, with an early childhood onset [1]. ASD is characterized by impairment in social communication and interactions, stereotyped and repetitive behaviours, altered sensory functions and variable degree of intellectual disability [1]. Family studies have clearly indicated the heritability of ASD, with more than 100 genes being associated with higher risk of developing autistic behaviours [2–4]. ASD-linked genes mainly encode for synaptic proteins, transcriptional regulators, and chromatin-remodelling factors [4, 5], suggesting that defects in the formation of synapses and in the establishment of neuronal circuits during the development of the central nervous system play a key role in ASD. Mutations in some of the ASD-linked genes are also associated with other syndromes sharing features with ASD, such as *FMR1* in fragile X syndrome, *UBE3A* in Angelman syndrome, *TSC1*/*TSC2* in Tuberous Sclerosis and *MECP2* in Rett syndrome [1, 6]. Other ASD-linked genes are not yet known to be causative of the disease, but their mutational status and/or expression levels were found to be altered in ASD patients.

Although the genetic contribution to the disease is well documented, ~75% of ASD patients lack a known genetic cause [7], suggesting that environmental factors can also play a crucial role in ASD onset [1]. Several non-genetic risk factors for the disease have been documented, including advanced parental age [8], neonatal hypoxia [9], premature birth, maternal stress and/or infections during pregnancy [10, 11]. Additionally, prenatal exposure to valproic acid (VPA), a drug used for treatment of epilepsy and other neuropsychological disorders, has been associated with an increased risk of ASD and represents a valuable animal model for the disease [6, 12, 13].

Gene expression coordination is crucial for proper brain development and establishment of neuronal circuits that govern motor and cognitive functions [4, 14]. Transcriptome analyses of ASD neural tissues have revealed distinct patterns of alterations. For instance, up-regulation of genes involved in glutamate signalling was detected in ASD brain tissues [15], which may underlie the excitatory/inhibitory imbalance observed in patients and animal models of the disease [16]. Moreover, genes involved in immune functions were also up-regulated in the brain of ASD patients [17], which probably indicates an increased inflammatory status and might be related to the enhanced risk of ASD observed after maternal infections during pregnancy [18]. On the other hand, ASD brains displayed reduced regional expression specificity with respect to control brains, which suggest loss of proper neuronal identity during cortical development [19].

While several brain regions have been suggested to be implicated in ASD [20], mounting evidence indicates a key role for the cerebellum. Indeed, altered development and function of this brain region represents the strongest non-genetic risk factor (>30-fold) for ASD, particularly when the defects occur within a defined perinatal window [18, 21]. The cerebellum is a highly folded and neuron-dense structure that has been classically involved in the control of motor functions. However, several studies have now clearly shown that the cerebellum is also involved in high order cognitive functions and social behaviours [22–24]. The posterior vermis, and particularly lobule VI and Crus I and II in lobule VII, display significant differences in ASD patients [25, 26]. Noteworthy, developmental defects in the cerebellum were reported in mice exposed to VPA during pregnancy. VPA-treated mice displayed premature migration of the granule cell (GC) precursors and decreased number of Purkinje cells (PCs) in the cerebellar cortex, which correlated with impaired synaptic functionality of PCs [27, 28]. These functional defects may result in widespread alterations of the brain connectivity network in ASD. Indeed, neural connections between the cerebellum and somatomotor, limbic and frontal cortical areas have been described and functional topography analyses revealed significant alterations of these circuits during both motor and non-motor tasks in ASD patients [29–33].

In the present study, we describe the gene expression programme that accompanies post-natal development of the mouse cerebellum and show that many ASD-associated genes undergo dynamic regulation. By using the VPA mouse model of ASD, we highlighted dysregulation of several ASD-linked genes in the developing cerebellum, which correlates with defective organization of the PCs in specific cerebellar subregions. Our results support the hypothesis that altered expression of ASD-linked genes in the developing cerebellum can contribute to the long-term behavioural features of the disease.

## Materials and methods

### Animal husbandry and experimental model

C57BL/6 mice were housed in cages with corncob bedding and maintained under standard laboratory conditions with an artificial 12-h light/dark cycle (lights on at 7:00 a.m. and off at 7:00 p.m.), controlled temperature and humidity. Mice took a standard diet with access to food and water ad libitum. Breeding, housing, maintenance, and behavioural tests were conducted according to the institutional guidelines of the Italian Institute of Health (protocol n. 750/2017-PR) in the animal facility of the Fondazione Santa Lucia IRCCS. Pregnant female mice were individually housed and treated at day 10.5 days post coitum (E10.5) with a single intra-peritoneal injection of VPA (P4543, Sigma-Aldrich) (500 mg/kg body weight dissolved in PBS). An equal volume of sterile saline solution was injected in control mice.

### RNA isolation and RT-PCR analyses

For RNA sequencing (RNA-Seq) analysis, cerebella from control male mice at different ages were isolated and maintained in RNA later stabilization reagent (76104, QIAGEN). Total RNA was extracted, and DNase treated using the RNAeasy Mini Kit (217004, QIAGEN). For validation assays, total RNA was isolated using the Trizol reagent (15596018, Invitrogen) according to the manufacturer’s protocol. Genomic was digested by RNase-free DNase (N2111, Roche). RNA purity and concentration were quantified using the NanoDrop 2000 UV spectrophotometer (Thermo Scientific). In all, 1 µg of total RNA was reverse-transcribed with random primers (11034731001, Sigma Aldrich) using M-MLV reverse transcriptase (M1705, Promega), according to the manufacturers’ instructions. 15 ng of cDNA were used as template for quantitative RT-PCR (qPCR) analysis. The qPCR reactions were performed using the LightCycler 480 System (Roche) with SYBR Green I Master Mix (04887352001, Roche) following the manufacturer’s instructions. The 2^−ΔCt^ method was applied to calculate differences in the gene expression using L34 gene for data normalization. Primers (Sigma) used in this study are listed in Supplementary Table 1. Male mice (*n* = 6 for both controls and VPA) were used for qPCR analyses.

### RNA-sequencing analysis

RNA was processed according to Illumina TruSeq Stranded mRNA sample preparation protocol using 2.500 ng per sample. Samples were paired-end sequenced at the Donnelly Sequencing Centre (University of Toronto) on Illumina NextSeq500 using high-output v2.0 300c chemistry. All RNA-Seq datasets were paired-end, 151-nucleotide length reads with 3 individual male mice for each sample type. Read depth was 25 M reads. Differentially expressed genes were identified using Deseq2 package in R Studio Software, with an adjusted cut-off of 0.05, an absolute log fold change of >1 required and a minimal expression level of 3 cRPKM. Clustering was done using the self-organizing maps algorithm and plotted using R Studio Software.

### Functional enrichment and computational analyses

Gene Ontology (GO) analyses were performed using the Panther Classification System [34] and the differentially expressed genes during cerebellum development were used as input. The same list of genes was then used to perform a Disease Ontology Enrichment Analysis on DisGeNet database [35] through Cluster Profiler package in R Studio Software. All analyses were performed using a background consisting of 12894 genes with the expression of at least cRPKM >2 in each one of the cerebellar samples and the following parameters: 0.05 *p* value, 0.2 *q*-value and Benjamini–Hochberg False Discovery Rate (FDR) as *p*-Adjusted Method. All the overlap analyses were performed using Venny 2.1.0 software. ASD susceptibility genes were obtained from the Simons Foundation Autism Research Initiative (SFARI) autism gene database [36], using the most up to date and high confidence SFARI gene list. The significance of the overlaps was assessed by hypergeometric test using the phyper function of R Stats Package in R Studio Software. For further analyses two different RNA-Seq datasets were used: GSE28521 [19] and GSE64018 [37]. Differentially expressed genes were obtained by raw count analysis using Deseq2 package in R Studio Software, as previously described [38]. Computational analysis of the distribution of gene length was calculated using the distance from Transcription Start Site to Transcription Start End of each gene with R Studio Software. GC content of the same gene groups was calculated using GenomicDistributions package in R Studio Software. All boxplots were plotted using R. Statistical significance refers to the differences between mean values and was calculated using the Student’s *t* test and applying the ‘Holm’ method for adjusted *p* values with ‘ggpubr’ package in R Studio Software.

### Western blot analysis

Total lysates from both control and VPA-treated male mice cerebella (*n* = 6) were obtained using RIPA buffer (50 mM Tris pH 7.4; 1% NP-40; 0.5% Na deoxycholate; 0.1% SDS; 150 mM NaCl; 1 mM EDTA) supplemented with protease inhibitor cocktail (P8340-5Ml, Sigma Aldrich), 0.5 mM Na_3_VO_4_ and 1 mM DTT. Lysates were incubated on ice for 20 min, briefly sonicated and centrifuged for 10 min at 13,000 rpm, 4 °C. Protein extracts were quantified by Bradford assay, resuspended at a final concentration of 20 μg and analysed by electrophoresis through 8–10% SDS-PAGE and blotted on a PVDF membrane (GE10600023, Amersham). Blots were firstly incubated with a blocking solution (5% non-fat dry milk in PBS) for 1 h at 25 °C and then with the following primary antibodies: mouse anti-β-ACTIN (sc-47778, Santa Cruz Biotechnology), rabbit anti-ROR-α (NR1F1, Novus), mouse anti-SHANK2 (NBP2-12914, Novus), rabbit anti-SHANK3 (NBP2-41171, Novus), mouse anti-GFAP (MAB-94373, Immunological Sciences). Antibodies were diluted 1:1000 in PBS/5% BSA solution and incubated overnight at 4 °C. After washing, blots were incubated with HRP-linked anti-rabbit and anti-mouse secondary antibodies (GE Healthcare) diluted 1:10,000 in PBS/5% non-fat dry milk. ECL signal was developed using Clarity Western ECL Blotting Substrate (Biorad) and acquired with the Alliance Q9 Advanced (Uvitec Cambridge).

### Immunocytochemistry and stereological estimations

To perform morphological analyses, brains from both control and VPA-treated male mice at post-natal day (P) 30 were collected (*n* = 4 for each group), fixed in 4% paraformaldehyde for 48 h and then washed in PBS. Whole brain tissues were dehydrated in a 30% sucrose solution. Serial sagittal free-floating slices (30 µm thickness) were cut using a cryostat (Leica CM 1850) and pre-treated with a blocking buffer solution (0.3% Triton X-100 in 3% horse serum in PBS) for 1 h. Every fourth sections were incubated with mouse monoclonal anti-CALBINDIN (CB) (C9848, Sigma, 1:1000) for 48 h at 4 °C, washed and incubated with a secondary biotinylated anti-mouse antibody (BA-9200, Vector Labs, 1:200) for 1 h at room temperature. The reaction was developed using the avidin–biotin peroxidase complex (ABC method, Vector Labs) and visualized using 3,3′-diaminobenzidine dissolved in 0.1 M phosphate buffer as a chromogen. Unbiased quantitative analysis of PCs in the cerebellar lobules VI, VII, VIII was performed using the optical fractionator stereological design and Stereo Investigator system with Stereo Investigator software Version 9, as previously described [39]. A MAC 6000 controller module, (MBF Bioscience, Williston VT, USA) was configured to interface with a Nikon Eclipse 80i microscope with a motorized stage and a digital colour camera (MBF Bioscience q imaging) with a Pentium II PC workstation. A three-dimensional optical dissector counting probe was applied to a systematic random sample of sites in the regions of interest at a magnification of 40x, (*x*, *y*, *z* dimension of 100 µm ×;100 µm × 10 µm, respectively, for cerebellar lobule VI; *x*, *y*, *z* dimension of 60 µm × 60 µm × 10 µm, respectively, for cerebellar lobule VII; *x*, *y*, *z* dimension of 80 µm × 80 µm × 10 µm, respectively, for cerebellar lobule VIII).

### Social approach, social novelty preference and grooming assays

Behavioural tests were performed on both adult male (*n* = 8 controls and *n* = 9 VPA) and female (*n* = 9 controls and *n* = 12 VPA) mice at P60. Subject animals were handled by the operator at least 24 h before testing and acclimatised for 30 min in the behavioural room. All behavioural tests were conducted between 10:00 a.m. and 3:00 p.m. and were video recorded while the operator was monitoring in blind the trials from a separate room. The three-chamber social behaviour test was performed as previously described [40], with small modifications [38]. The movements of testing mice were recorded using a digital video camera placed on top of the cage and tracked using the Ethovision XT software. During the entire duration of the three-chamber social behaviour test, also self-grooming duration and frequency were evaluated to assess the presence of repetitive or stereotypical actions [41].

### Quantification and statistical analysis

All quantitative data are expressed as the mean ± standard deviation (SD) or standard error of the mean (SEM), as indicated in the figure legends. Two-tailed Student’s *t* test and one-way and two-way analysis of variance (ANOVA) followed by Bonferroni’s multiple comparison post-test were performed using Graphpad Prism v7. *p* Value < 0.05 was considered significant. For behavioural tests, we carried out a power analysis based on literature data [40], which yielded *n* = 7. Thus, we used a minimum of eight mice for each experimental group. All behavioural tests were performed by a blinded experimenter.

## Results

### Post-natal cerebellum development is accompanied by dynamic gene expression reprogramming

The cerebellum is one of the first brain structures to begin cell differentiation and among the last to complete maturation [42]. In the mouse, most of the crucial morphological and molecular differentiative events, including foliation and establishment of synaptic contacts, occur after birth [18, 42]. Given the higher incidence of ASD in males [43], we set out to investigate the transcriptome changes that accompany post-natal development of the cerebellum by performing high-throughput RNA-Seq analyses (GSE133711) [38] in male mice. We selected three key developmental stages: P1, when PCs start to mature and GCs proliferate in the external granular layer (EGL); P10, when post-mitotic GCs establish functional connections with PCs during their migration into the internal granular layer (IGL); P30, when neurons have completed their differentiation and the cerebellum has acquired its mature morphological and functional stages [42, 44, 45].

**Fig. 1:**
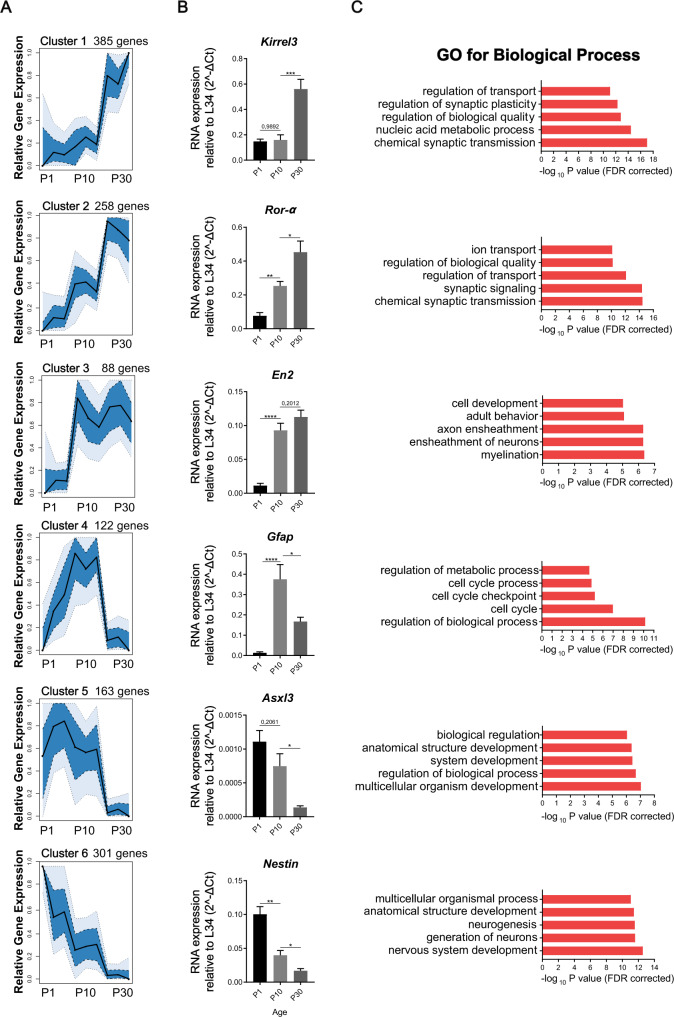
A dynamic gene expression programme characterizes post-natal cerebellum development in mice. **A** Temporal clustering based on the relative gene expression at P1, P10, and P30 of the 1316 developmental-regulated genes in the mouse cerebellum. Polygon plots show the median values as a black line. The dark blue represents the 0.25–0.75 quantiles and the light blue 0.1–0.9 percentiles. The expression values were normalized by maximum expression value. **B** Analysis by qPCR of one representative developmental regulated gene for each cluster. Data are expressed as: mean ± SEM, *n* = 6, *p value ≤ 0.05; **p value ≤ 0.01; ***p value ≤ 0.001; ****p value ≤ 0.0001; number = not significant, test = one-way ANOVA Tukey’s multiple comparisons. **C** GO analysis for Biological Process for each cluster of cerebellum development regulated genes. Bar length is measured by −log10 *p* value (FDR corrected).

Bioinformatic analyses of the RNA-Seq data highlighted extensive transcriptional changes occurring during cerebellar development, with 1316 genes being differentially expressed from P1 to P30 (10.2% of expressed genes; FDR < 0.05). Analysis of expression patterns by an unsupervised machine learning approach (self-organized maps) uncovered six temporal clusters, each one with a distinct expression profile during the P0–P30 time window (Fig. 1A). Differentially expressed genes were either progressively up-regulated (19.6% of total; Cluster 2) or down-regulated (22.9% of total; Cluster 6) during development (Fig. 1A, B). GO analysis showed that progressively up-regulated genes (Cluster 2) were enriched for synaptic processes, whereas progressively downregulated genes (Cluster 6) were related to neurogenesis (Fig. 1C). Other synaptic genes were specifically up-regulated between P10 and P30 (29.3% of total; Cluster 1) (Fig. 1A–C), likely reflecting synapse specification at later phases of differentiation. Likewise, some developmental genes were also downregulated only at this later time-window (12.4% of total; Cluster 5). Interestingly, cell cycle and cell cycle checkpoint genes were sharply up-regulated only at P10 (9.3% of total; Cluster 4), when a clear switch between the proliferative and differentiative phases of granule cells occurs in the EGL [44, 45]. On the other hand, genes involved in axonal myelination (6.7% of total; Cluster 3) were up-regulated at P10 and their expression persisted throughout developmental completion (Fig. 1A–C). Analysis by qPCR analysis of arbitrarily selected genes (*n* = 18) from the different clusters yielded >80% validation rate (Fig. 1B and Supplementary Fig. 1A, B), indicating the reliability of the RNA-Seq and bioinformatics analyses. These results highlight the existence of a highly dynamic gene expression network that accompanies, and likely determines, the different stages of post-natal cerebellar development.

### ASD-linked genes are dynamically regulated during cerebellar development

**Fig. 2:**
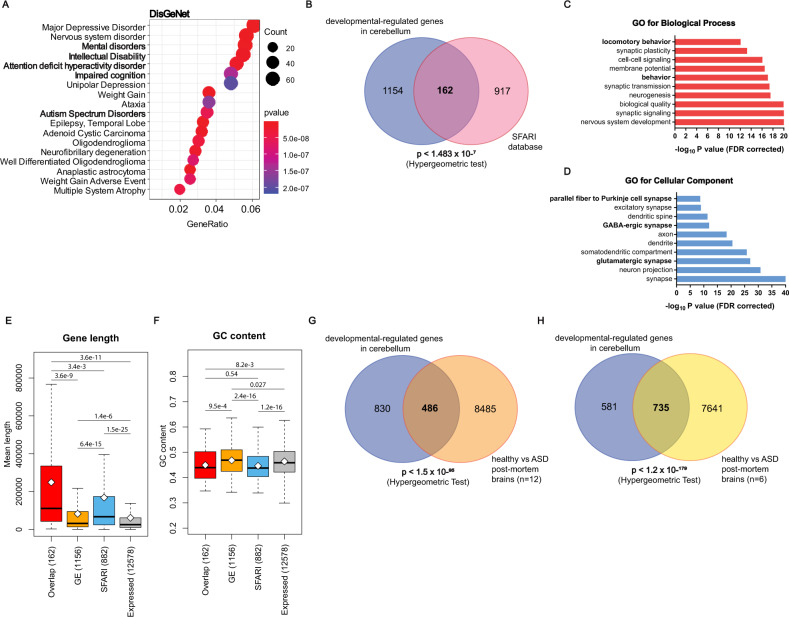
Developmental regulated genes are significantly associated with ASD. **A** Disease Ontology Enrichment analysis of the 1316 cerebellar developmental regulated genes. The dot size in the graph is proportional to the number of genes in each category, while the dot colour represents the enrichment grade, with red indicating high enrichment score and blue indicating low enrichment score. Gene ratio is the percentage of the 1316 genes in the given GO term. **B** Venn diagram showing the overlap between the genes differentially expressed during cerebellar development (blue) and the genes annotated in the SFARI database (pink). The population of expressed genes in cerebellum was used as background for the statistical analysis. **C**, **D** Analysis of the Biological Processes (**C**) and Cellular Components (**D**) that are enriched among the 1316 developmental regulated genes. Bars length represents that −log10 *p* value (FDR corrected). **E**, **F** Boxplot representing the distribution of gene length (**E**) and GC content (**F**) among the: 162 genes regulated during cerebellum development and overlapping with SFARI database; genes regulated in the developing cerebellum (*n* = 1156); genes annotated in SFARI database but not regulated during cerebellum development (*n* = 882); total genes expressed in the cerebellum (*n* = 12,578). Whiskers indicate 1.5 interquartile range. *p* Values refer to the differences between mean values and was estimated using the Student’s *t* test. **G**, **H** Venn diagrams showing the overlap between the genes differentially expressed during cerebellar development (blue) and those differentially regulated between either 12 healthy and 12 ASD post-mortem brains (**E**, orange) or between 6 healthy and 6 ASD post-mortem brains (**F**, yellow) in two independent studies.

Altered cerebellar development is a risk factor for neurodevelopmental diseases, including ASD [18, 21]. To investigate whether the transcriptional programme that accompanies the cerebellar post-natal development includes modulation of genes associated with neurodevelopmental diseases, we carried out enrichment analyses. By querying DisGeNet, a platform containing one of the largest public collections of genes and variants associated to human diseases [35], we observed that genes undergoing differential expression during cerebellar development are highly associated with mental disorders and intellectual disability, including ASD (Fig. 2A). Likewise, analysis of the Human Phenotype Ontology, a standardized vocabulary of phenotypic abnormalities encountered in human diseases [46], confirmed that the transcriptional programme governing cerebellum development is significantly enriched for genes associated with ASD and other cognitive impairments (Supplementary Fig. 2A). Furthermore, there is a significant overlap (*p* < 1.48e^−7^, Hypergeometric test) between the developmental-regulated genes and those annotated in the SFARI database as directly linked to ASD, with 162 genes in common between the two datasets (Fig. 2B). GO analyses for Biological Processes and Cellular Components of these 162 genes highlighted terms related to nervous system development, synaptic signalling and regulation of synaptic plasticity (Fig. 2C, D), all processes that are dysregulated in ASD [2–4]. A significant overlap with ASD-linked genes (*n* = 175; *p* < 7.02e^−14^) was also found by analysing the Autism Gene Database (AutDB) (Supplementary Fig. 2B), a public database for manual annotation and visualization of genes linked to ASD [47]. Notably, 159 of the genes that are dynamically regulated during cerebellar development were annotated in both ASD databases (Supplementary Fig. 2C and Supplementary Table 2). Of particular interest for the establishment of the cerebellar circuitry are genes involved in synapses between parallel fibres and PCs dendrites (*Grm4*, *Grin1*, *Grid2*, *Ctnna2*, *Atp2b2*, *Cbln1*, *Cntn6*, *Scn8a, Cadps2*), a process that occurs during migration of GCs from the EGL to the IGL [45].

Computational analyses indicated that the 162 ASD-associated genes that are dynamically regulated during cerebellar development (Overlap group) are significantly larger than other developmental-regulated genes (GE group) or other genes expressed in the cerebellum (Fig. 2E). Although larger size was also common to the rest of SFARI-annotated genes, those undergoing dynamic expression changes were significantly larger (Fig. 2E). Moreover, we found that SFARI-annotated genes are characterized by significantly lower GC content than other genes expressed in the cerebellum (Fig. 2F). However, there was no significant difference between the 162 genes undergoing dynamic changes in cerebellar development and the rest of SFARI-annotated genes (Fig. 2F). The lower GC content may be due to the larger size, as introns are generally GC-depleted with respect to exons and length of genes is generally associated with introns length [48].

To test whether developmental-regulated genes were also associated with other psychiatric syndromes, we queried datasets of genes associated with schizophrenia [49] and bipolar disorder [50, 51]. However, this analysis indicated no significant overlap (Supplementary Fig. 2D, E). In further support of a specific link between gene expression modulation during post-natal cerebellar development and ASD, analysis of genes differentially expressed in two studies of post-mortem ASD brains (GSE28521, GSE64018) [19, 37] indicated a significant overlap (Fig. 2G, H). These analyses indicate that expression of a large subset of genes mutated or differentially expressed in ASD is dynamically regulated during post-natal cerebellar development.

### Fetal exposure to VPA induces ASD-associated behaviours and cerebellar cortical alterations

**Fig. 3:**
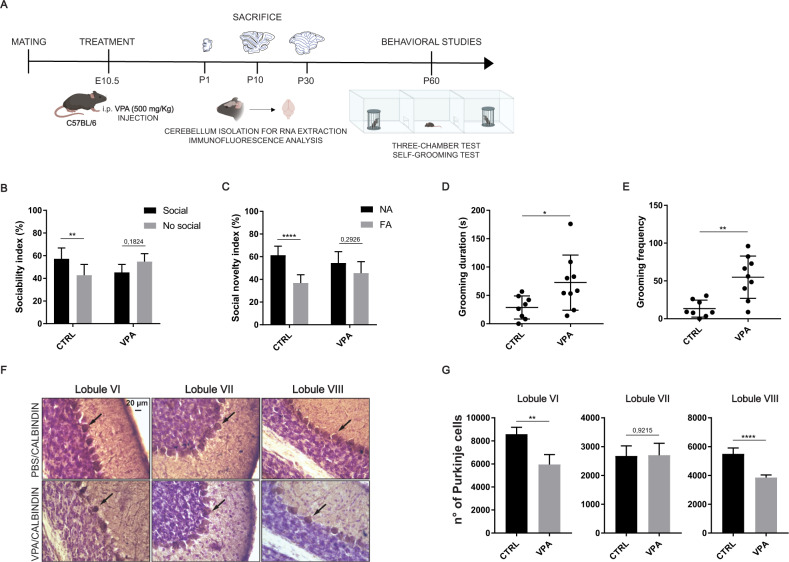
Behavioural and morphological alterations in the VPA-treated mice. **A** Experimental design used to develop the VPA model of ASD. **B** Sociability index in male Ctrl (*n* = 8) and VPA (*n* = 9) mice measured by the percentage of time spent in close interaction with the animal or the object over the sum of the two interactions. **C** Social novelty index in male Ctrl (*n* = 8) and VPA (*n* = 9) mice measured by the percentage of time spent in close interaction with the familiar animal (FA) or a novel animal (NA). Data are expressed as: mean ± SD, *p value ≤ 0.05; **p value ≤ 0.01; ***p value ≤ 0.001; ****p value ≤ 0.0001; number = not significant, statistical test = two-way ANOVA Tukey’s multiple comparisons. **D** Grooming duration in male Ctrl (*n* = 8) and VPA (*n* = 9) mice measured by the total time (seconds) spent in grooming. **E** Grooming frequency in male Ctrl (*n* = 8) and VPA (*n* = 9) mice measuring the number of times mice had grooming. Data are expressed as: mean ± SD, *p value ≤ 0.05; **p value ≤ 0.01; ***p value ≤ 0.001; ****p value ≤ 0.0001; number = not significant, statistical test = unpaired *t* test. **F** Calbindin immunostaining of Ctrl and VPA mice cerebella (Lobules VI, VII, and VIII) at P30. Black arrows indicate the PCs. **G** Quantitative analysis of the number of PCs in the indicated cerebellar lobules as determined by stereotaxic analysis. Data are expressed as: mean ± SD, *p value ≤ 0.05; **p value ≤ 0.01; ****p value ≤ 0.0001; number = not significant, test = unpaired *t* test.

Exposure to VPA during pregnancy was shown to increase the risk of autism in children [52]. Likewise, rodents exposed to VPA during fetal development display autistic features after birth [6, 13]. Thus, we employed the VPA model to test whether cerebellar expression of ASD-linked genes is dysregulated in mice affected by the disease. Pregnant females were treated with a single peritoneal injection of VPA at embryonic day 10.5 (E10.5), a protocol that was previously shown to alter synaptic transmission in the cerebellar cortex [27, 28]. After birth, cerebella were isolated from control (saline) and VPA-treated mice at P1, P10 and P30 for RNA and protein analysis (Fig. 3A). In parallel, littermate mice were analysed at P60 by behavioural tests related to ASD (Fig. 3A).

First, we assessed the performance of VPA-treated mice in the three-chamber sociability test [40]. Analysis of the exploratory ability (total distance and walking time for each mouse) during the two phases of the test, the sociability phase (Soc1) and the social novelty phase (Soc2) respectively, indicated no impairment of locomotion in both female and male VPA-treated mice (Supplementary Fig. 3A). However, unlike control mice, VPA-treated mice displayed a social interaction deficit, as they did not exhibit preference for the social (mouse) with respect to the non-social (object) cue during the Soc1 phase of the test (Fig. 3B). Furthermore, in the following Soc2 phase, VPA-treated mice failed to spend more time with the novel mouse compared to the familiar one (Fig. 3C). Similar results were also obtained with female VPA-treated mice (Supplementary Fig. 3B, C). Next, we evaluated the self-grooming activity of mice, a test used to assess repetitive behaviour in mice [41]. VPA-treated male and female mice displayed increased self-grooming duration (seconds) and the frequency (number of times) compared to control mice (Fig. 3D, E and Supplementary Fig. 3D, E). Collectively, these analyses confirmed the efficacy of a single VPA injection during fetal development to induce ASD-related social and repetitive behaviours in the experimental mice.

Aberrant cerebellar development is associated to ASD [18, 21]. In particular, ASD individuals were reported to exhibit alterations of lobules VI and VII [53, 54] and a decrease in the number of PCs [55]. Importantly, progressive loss of PCs was also observed in VPA-treated mice [28]. To test whether this feature was recapitulated in our experimental model, the number of PCs was evaluated in the central cerebellar lobules VI-VIII at P30. Immunohistochemical staining for the PC marker calbindin and stereological analyses of cerebellar sections showed a significant decrease of PCs specifically in lobules VI and VIII of VPA-treated mice compared to controls, whereas no differences were observed in lobule VII (Fig. 3F, G). These results confirmed morphological alterations of the cerebellar cortex in our ASD mouse model.

### Fetal exposure to VPA dysregulates the expression of ASD-linked genes in the cerebellar cortex

**Fig. 4:**
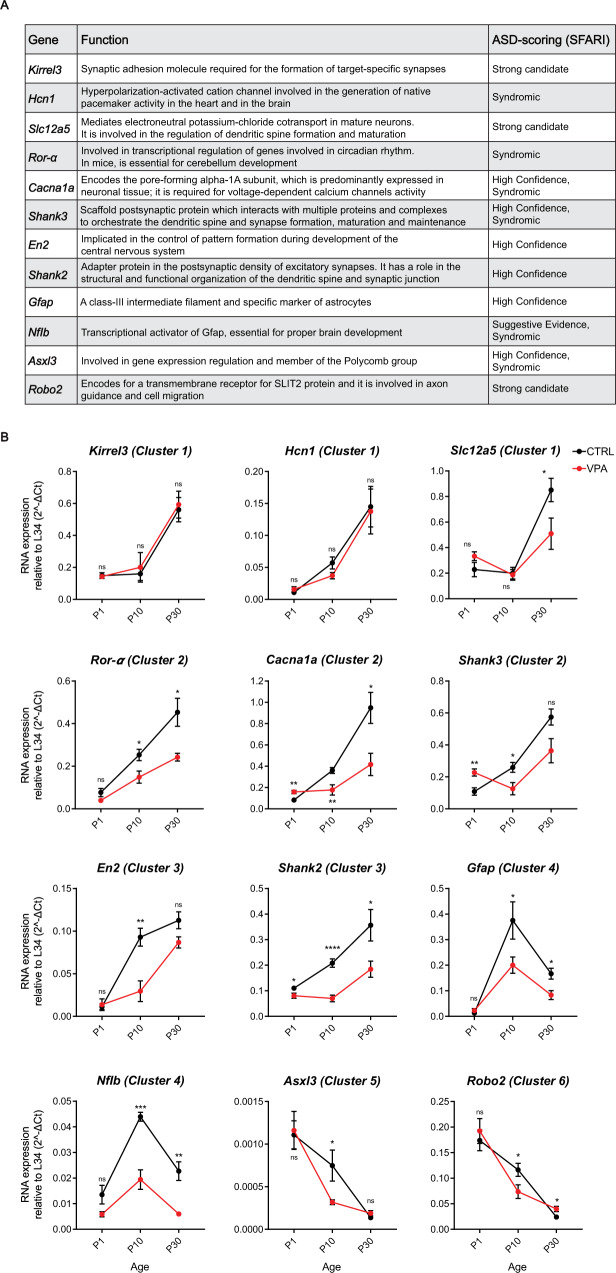
Expression of ASD-associated genes is altered in VPA-treated mice. **A** List of the genes selected for their strong association with ASD. **B** Analysis by qPCR of the genes described in **A** in Ctrl and VPA mice at P1, P10, and P30. Data are expressed as: mean ± SEM, *n* = 6, *p value ≤ 0.05; **p value ≤ 0.01; ***p value ≤ 0.001; ****p value ≤ 0.0001; number = not significant; statistical test = two-way ANOVA Tukey’s multiple comparisons.

ASD behavioural features, as altered information processing and cognitive impairments, are caused by alterations in multiple processes, including progenitor cell proliferation, neuronal differentiation, and synapses formation [56, 57]. Since the expression of ASD-linked genes that are involved in synaptic functions and neurogenesis is dynamically regulated during post-natal cerebellar development (P1-P30), we asked whether VPA affected their expression pattern. Following the SFARI criteria for the overall strength of evidence, we selected genes that are either strongly associated with ASD (i.e., *Ror-α*, *Shank2*, *Shank3* and *Robo2*) or whose alterations have been recently associated with ASD [58–68] (Fig. 4A). Analysis by qPCR revealed that the developmental pattern of expression of most of these ASD-linked genes was altered in the cerebellum of VPA-exposed mice. Some of them, such as *Slc12a5* (cluster 1), *Ror-α* (cluster 2), *Gfap* and *Nflb* (cluster 4), displayed an overall reduction in expression, while maintaining the dynamic pattern observed in control mice (Fig. 4B). On the other hand, expression of other ASD-linked genes also displayed an altered pattern (Fig. 4B), with either delayed increase (cluster 2 genes *Cacna1a*, *Shank3* and cluster 3 genes *En2, Shank2*) at P30 or anticipated decrease at P10 (cluster 5 *Asxl3* and cluster 6 *Robo2* genes). Only two of the tested genes (cluster 1 *Kirrel3* and *Hcn1*) were not affected by VPA treatment in the developing cerebellum (Fig. 4B). Collectively, these results indicate that fetal exposure to VPA exerts a long-range impact on the expression of ASD-linked genes, particularly at P10 (Supplementary Fig. 4), when critical developmental processes involved in cortical development and synapse formation occur.

### VPA-induced gene expression changes reflect into reduced protein expression in the cerebellar cortex

**Fig. 5:**
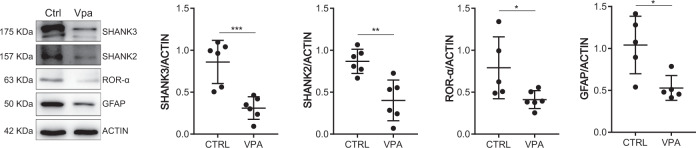
SHANK3, SHANK2, ROR-α and GFAP proteins are downregulated in VPA mice at P10. Representative western blot images of the indicated proteins in Ctrl and VPA cerebella at P10 (left panel) and relative densitometric analysis (right panel). Data are expressed as: mean ± SD, *p value ≤ 0.05; **p value ≤ 0.01; ***p value ≤ 0.001; ****p value ≤ 0.0001; number = not significant, test = unpaired *t* test.

To test whether the observed changes at transcript level were functionally relevant, we evaluated the expression of a subset of encoded proteins at P10. Western blot analysis of cerebellar extracts confirmed the inhibitory effect of VPA treatment at the protein level (Fig. 5). For instance, the expression of scaffolding proteins SH3 and multiple ankyrin repeat domains proteins 2 (SHANK2) and 3 (SHANK3), which are both strongly linked to syndromic and idiopathic ASD [63], was significantly reduced in VPA-treated cerebella with respect to control cerebella of the same developmental stage (Fig. 5). Likewise, the Retinoic acid-related Orphan Receptor alpha (ROR-α), which is associated with both syndromic and genetically-linked ASD risk [61], and the Glial Fibrillary Acidic Protein (GFAP), whose expression is altered in subject with ASD [65], were significantly decreased at P10 in VPA-treated mice. Collectively, our data indicate that gene expression changes induced by fetal exposure to VPA impair the expression of key ASD-associated genes in the developing cerebellum.

## Discussion

Post-natal development of the cerebellum is emerging as a very sensitive period for the acquisition of cognitive functions and as a critical vulnerability associated with ASD [18, 21, 69]. In this study, we elucidated a dynamic gene expression programme that accompanies mouse cerebellum development in the first month after birth. Transcriptomic analyses at three crucial time points of cerebellar development [42, 44, 45] revealed six temporal clusters of gene expression regulation that affect different processes in cerebellar development, from neurogenesis to synapse functional specification. The identified patterns of expression were coherent with the stage of development. Indeed, while genes encoding for synaptic proteins increase in expression between P10 and P30 (Clusters 1 and 2), concomitantly with establishment and functional specification of the synapse in the cerebellum, an inverse regulation was observed for genes related to neurogenesis and nervous system development. Other patterns were less obviously related to the developmental stage. For instance, genes related to cell cycle and cell cycle checkpoints were sharply up-regulated only at P10 (Cluster 4) and declined afterward. This specific increase at P10 is not a direct consequence of augmented proliferation at this stage, but it may relate to the need of cells to switch from proliferation to differentiation and to migrate from the EGL to the IGL [45]. On the other hand, genes involved in axonogenesis and myelination (Cluster 3) are also upregulated at P10 but remain highly expressed until cerebellar development is complete. Thus, P10 appears as a critical stage for mouse cerebellar development and perturbation of gene expression at this timepoint may disrupt the morphogenetic programme required for the assembly of a proper cerebellar cortex. Notably, Cluster 3 was also enriched for genes related to adult behaviour (*Shank2*, *Eps8*, *Prex2*, *Adam22*, *Cntn2*, *Nos1*, *Scn8a*, *Kncnj10*), a feature that is fully achieved later in life. This early expression of behaviour-related genes may explain why perturbation of gene expression in the cerebellum in this time window has been frequently associated with long-term effects and increased risk of neurodevelopmental disorders, including ASD [18, 21].

The gene expression programme that accompanies post-natal cerebellar development is highly relevant for ASD. In support of this notion, we found a significant overlap between developmental-regulated genes and those linked to ASD in two independent databases (SFARI and AutDB) [36, 47], but not with genes associated to other psychiatric disorders associated with cognitive dysfunction. Moreover, the genes dynamically regulated during cerebellar development were also found dysregulated in two studies that compared ASD brains with healthy brains [19, 37], suggesting that developmental-regulated genes are relevant for the disease. To further assess the relevance for ASD of this developmental-regulated programme, we employed the VPA mouse model, a non-genetic model of autism [6, 13]. Exposure to VPA during pregnancy is a risk factor for ASD in children [12], while treatment with VPA at early stages of development was shown to induce ASD-associated features in mice [6, 13]. Our behavioural tests confirmed the efficacy of a single injection of VPA at E10.5 in inducing social impairment and stereotyped repetitive behaviours in the experimental mice evaluated at the adult stage (P60). Furthermore, these mice displayed morphological alterations in cerebellar regions previously shown to be relevant for ASD [25, 26]. More importantly, several genes strongly associated with ASD are dysregulated in the cerebellum of VPA mice. In the case of *Shank3*, its conditional deletion in the cerebellum was shown to alter neuronal plasticity, a defect that was associated with altered function of both PCs and GCs [70]. On the other hand, the direct contribution to ASD traits of many other ASD-linked genes identified here as dynamically regulated during cerebellar development in currently unknown. However, since these genes are involved in synapse formation and function, alterations of their expression during cerebellar development are likely to impact on the physiology of the cerebellar circuits. Thus, our studies strengthen the notion that perinatal cerebellar development is a sensitive period for the establishment of correct functional connections, whose disruption may result in ASD-associated phenotypes.

Expression of genes in all clusters, except for Cluster 1, were affected in the cerebella of VPA mice, with a trend that suggested a delayed temporal expression. Notably this dysregulation leads to significant downregulation of the corresponding proteins, thus possibly mimicking the loss-of-function effect observed in ASD samples harbouring inactivating mutations in these genes. For instance, the simultaneous downregulation of both *Shank2* and *Shank3* proteins was recently shown to impair social memory [71], a feature strictly associated with autism. impairment. Likewise, dwnregulation of ROR-α could also result in dysregulation of synaptic function, as this transcription factor is highly expressed in PCs, guides the expression of proteins required for transducing the glutamatergic signal from GCs and its mutation is associated with acquisition of ASD traits [72].

Previous studies indicated that VPA modulates the expression of several ASD-linked genes in the mouse neocortex [73] and in human cortical organoids in vitro [74]. Our study is in line with these previous observations and support the evidence that exposure to environmental factors in utero can also exerts long-range effects on the gene expression programme of the cerebellum, impacting on multiple genes related to ASD and possibly contributing to the onset and progression of the disease.

## Supplementary information

Supplemental Tables and Figures
