# Supplementary material for: Fetal exposure to valproic acid dysregulates the expression of autism-linked genes in the developing cerebellum
Source: Transl Psychiatry. 2023 Apr 5;13:114. doi: 10.1038/s41398-023-02391-9 (PMC10076313; doi:10.1038/s41398-023-02391-9)
Supplement: Supplementary file 1 — Supplemental Tables and Figures [file 41398_2023_2391_MOESM1_ESM.pdf]

Supplementary Table 1. Oligos used in this paper.

Cluster/Type	Gene	Sequence	Amplicon lenght (bp)
Normalizer	L34_Fw	GGTGCTCAGAGGCACTCAGGATG	144
	L34_Rev	GTGCTTTCCCAACCTTCTTGGTGT	
Cluster 1	KIRREL3_Fw	TCGAGACGGCAAACGAGAA	88
	KIRREL3_Rev	GGCTCGGCACACAATACTCT	
Cluster 1	SLCA25_Fw	GGGCAGAGAGTACGATGGC	111
	SLCA25_Rev	TGGGGTAGGTTGGTGTAGTTG	
Cluster 1	HCN1_Fw	CTCAGTCTCTTGCGGTTATTACG	91
	HCN1_Rev	TGGCGAGGTCATAGGTCAT	
Cluster 1	STXBP1_Fw	AGGTGCTACTGGATGAGGACGA	127
	STXBP1_Rev	TCGCCAGTGTTCATCCTCTTGC	
Cluster 2	CACNA1A_Fw	GAAGTGAGTCCCCTGTCTGC	83
	CACNA1A_Rev	CACACCGACTTGGTAGGCTT	
Cluster 2	SHANK3_Fw	GCCTTCCCTGCACTCCAATA	127
	SHANK3_Rev	GCTTGTGTCCAACCTTCACG	
Cluster 2	RORα_Fw	CTTCCCCTACTGTTCCTTCACC	201
	RORα_Rev	CACATCACCTCTCTCTGCTTGT	
Cluster 3	SHANK2_Fw	ACGGTGGTCCTGCAAAAGAA	118
	SHANK2_Rev	GGTACTGCAGGGCTGGAAAT	
Cluster 3	EN2_Fw	AATCAAGAAAGCCACGGGCA	109
	EN2_Rev	CTACTCGCTGTCCGACTTGC	
Cluster 4	GFAP_Fw	TTTGCAGACCTCACAGACGC	84
	GFAP_Rev	TTGGCGGCGATAGTCGTTAG	
Cluster 4	NFIB_Fw	GGGACTAAGCCCAAGAGACC	63
	NFIB_Rev	GTCCAGTCACAAATCCTCAGC	
Cluster 4	SMC3_Fw	CTTGTGTGGAAGTCACTGCTGG	125
	SMC3_Rev	AGGCAGGAAAGTCACCTCTCCA	
Cluster 4	SNCA_Fw	CACTGGCTTTGTCAAGAAGGACC	104
	SNCA_Rev	CATAAGCCTCACTGCCAGGATC	
Cluster 5	EZH2_Fw	CTAATTTGGTACTTACTACGATAACTTT	87
	EZH2_Rev	ACTCTAAACTCATACACCTGTCTACAT	
Cluster 5	ASXL3_Fw	AAACAGCGACTGGCAGAAG	124
	ASXL3_Rev	TCTCTCCATAAAACCTCTCAAAGA	
Cluster 6	NESTIN_Fw	GAGTTCTCAGCCTCCAGCAG	129
	NESTIN_Rev	GCAGGAGAAGCAGGGTCTAC	
Cluster 6	ROBO2_Fw	CGAGCTCCTCCACAGTTTGT	136

Supplementary Table 2. List of the 159 genes regulated during mouse cerebellum development and annotated in both SFARI and AutDB databases.

Adamts18	*Dagla*	*Grin1*	*Nrp2*	*Stx1a*
Adarb1	*Dapk1*	*Grip1*	*Pcdh10*	*Stxbp1*
Ank2	*Dcx*	*Grm1*	*Pcdh19*	*Syn1*
Anks1b	*Dgkz*	*Grm4*	*Pcdh8*	*Syn2*
Apc	*Dixdc1*	*Grm5*	*Pdk2*	*Syncrip*
Arhgap33	*Dlg1*	*Gsn*	*Ppp1r1b*	*Syne1*
Arhgap5	*Dlgap1*	*Gstm1*	*Prickle2*	*Tanc2*
Arhgef10	*Dock4*	*Hcn1*	*Prune2*	*Tspan7*
Arhgef9	*Dpp10*	*Hmgn1*	*Psd3*	*Unc80*
Arnt2	*Dpysl3*	*Hsd11b1*	*Pvalb*	*Vash1*
Aspm	*Dscam*	*Il16*	*Pxdn*	*Zbtb20*
Asxl3	*Ebf3*	*Iqgap3*	*Rapgef4*	
Atp1a1	*Eef1a2*	*Iqsec2*	*Rbfox1*	
Atp1a3	*Efr3a*	*Itpr1*	*Rbms3*	
Atp2b2	*En2*	*Kat2b*	*Rgs7*	
Bcas1	*Ephb2*	*Kcnc1*	*Rims1*	
Cacna1a	*Eps8*	*Kcnd2*	*Rims3*	
Cacna1e	*Erbb4*	*Kcnj10*	*Rit2*	
Cacna1g	*Ermn*	*Kcnj12*	*Rnf38*	
Cacna1h	*Fabp3*	*Kcnt1*	*Robo1*	
Cacna1i	*Fabp5*	*Kif21b*	*Robo2*	
Cadps2	*Fabp7*	*Kirrel3*	*Rora*	
Camk2a	*Fat1*	*Klc2*	*Scn1a*	
Camk2b	*Fbn1*	*Mal*	*Scn8a*	
Camk4	*Foxp2*	*Mapk8ip2*	*Sez6l2*	
Cbln1	*Frmpd4*	*Mark1*	*Shank2*	
Cdh11	*Gabra1*	*Mcm6*	*Shank3*	
Cdh13	*Gabra3*	*Megf11*	*Slc1a2*	
Cecr2	*Gad1*	*Meis2*	*Slc24a2*	
Cgnl1	*Galnt13*	*Myo16*	*Slc4a10*	
Chd5	*Gap43*	*Nefl*	*Slc6a1*	
Clstn3	*Gatm*	*Negr1*	*Slitrk5*	
Cnksr2	*Glra2*	*Neo1*	*Smarca2*	
Cnr1	*Gria1*	*Nfib*	*Smc3*	
Cntn5	*Grid2*	*Nfix*	*Snap25*	
Cntn6	*Grid2ip*	*Nos1*	*Spast*	
Ctnna2	*Grik3*	*Nr1d1*	*St8sia2*	

Supplementary Figure legends

Suppl. Fig. 1. Validation of the transcriptome analysis in post-natal cerebellum. Selected genes of each cluster were analysed by qPCR at P1, P10 and P30 in Ctrl and VPA cerebella. Data are expressed as: mean ± SEM, n=6, *p value≤0.05; **p value≤0.01; ***p value≤0.001; ****p value≤0.0001; number=not significant, test= One Way ANOVA Tukey's multiple comparisons. A) Validated genes. B) Not-validated genes.

Suppl. Fig. 2. Gene ontology analyses of the developmental regulated genes and their overlap with genes annotated in the AutDB database. A) Human Phenotype Ontology Enrichment analysis of the developmental regulated genes described in Fig. 1. B) Venn diagram showing the overlap between the developmental regulated genes (blue) and those annotated in the AutDB database (green). C) Venn diagram showing the overlap between the developmental regulated genes (blue), those annotated in the SFARI (pink) and AutDB databases (green). D) Venn diagram showing the overlap between the differentially expressed genes during cerebellar development (blue) and schizophrenia associated genes (purple). E) Venn diagram showing the overlap between the differentially expressed genes during cerebellar development (blue) and bipolar disorder associated genes (purple).

Suppl. Fig. 3. Behavioural features of VPA-treated mice. A) Total exploration time in Ctrl and VPA mice during the Soc 1 (left panel) and Soc 2 (right panel) phases. B) The bar graph represents the quantitative analysis of the sociability index, expressed as percentage of social versus non-social, in female Ctrl (n=9) and VPA (n=12) mice. C) The bar graph represents the quantitative analysis of the social novelty index, expressed as percentage of novel animal versus familial animal, in female Ctrl (n=9) and VPA (n=12) mice. D,E) Dot-plot representation of the total grooming duration (D) measured by the total time (seconds) spent in grooming and grooming frequency measuring the number of times mice had grooming (E), in female Ctrl (n=9) and VPA (n=12) mice.

Suppl. Fig. 4. Gene expression alterations in ASD mouse model. A) Analysis by qPCR of selected ASD-associated genes at P1, P10 and P30 in Ctrl and VPA mice. Data are expressed as: mean ± SEM, n=6, *p value≤0.05; **p value≤0.01; ***p value≤0.001; ****p value≤0.0001; number=not significant, test=Two Way ANOVA Tukey's multiple comparisons.

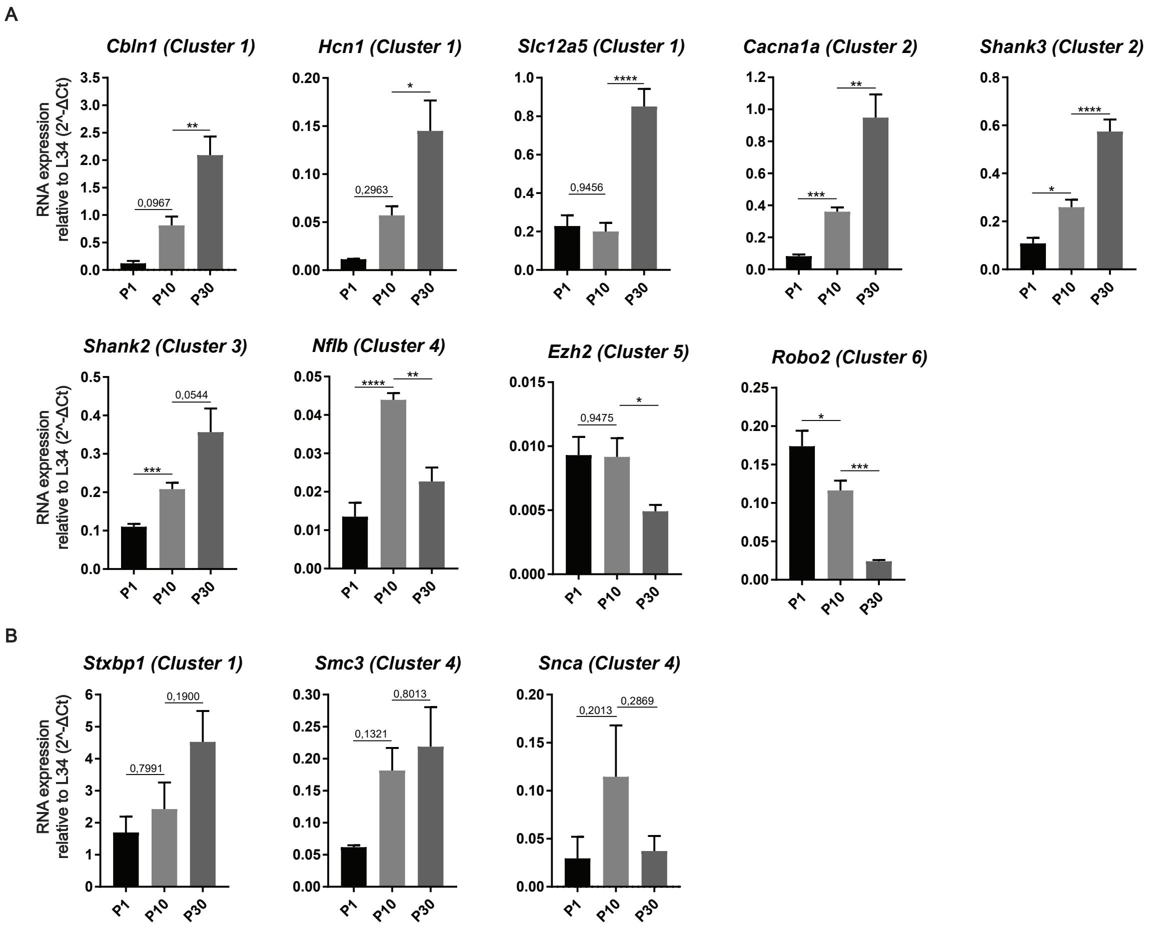

A
Cbln1 (Cluster 1)
Hcn1 (Cluster 1)
Slc12a5 (Cluster 1)
Cacna1a (Cluster 2)
Shank3 (Cluster 2)
Shank2 (Cluster 3)
Nflb (Cluster 4)
Ezh2 (Cluster 5)
Robo2 (Cluster 6)
RNA expression relative to L34 (2^-ΔCt)
P1
P10
P30
0,0967
0,2963
0,9456
0,0544
0,9475
B
Stxbp1 (Cluster 1)
Smc3 (Cluster 4)
Snca (Cluster 4)
0,7991
0,1900
0,1321
0,8013
0,2013
0,2869

Suppl. 1

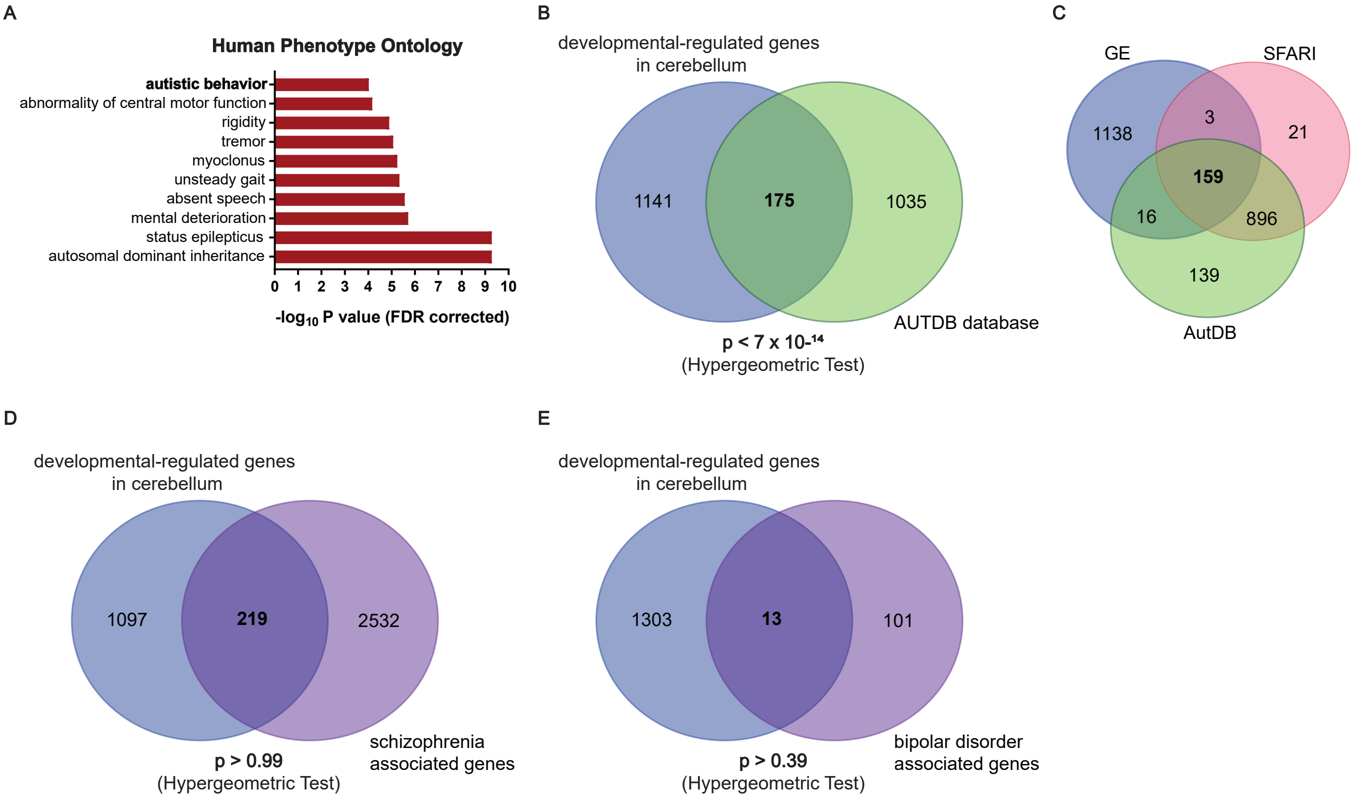
A
Human Phenotype Ontology
autistic behavior
abnormality of central motor function
rigidity
tremor
myoclonus
unsteady gait
absent speech
mental deterioration
status epilepticus
autosomal dominant inheritance
0 1 2 3 4 5 6 7 8 9 10
-log$_{10}$ P value (FDR corrected)
B
developmental-regulated genes in cerebellum
1141
175
1035
AUTDB database
p < 7 x 10-^{14}
(Hypergeometric Test)
C
GE
SFARI
1138
3
21
159
16
896
139
AutDB
D
developmental-regulated genes in cerebellum
1097
219
2532
schizophrenia associated genes
p > 0.99
(Hypergeometric Test)
E
developmental-regulated genes in cerebellum
1303
13
101
bipolar disorder associated genes
p > 0.39
(Hypergeometric Test)

Suppl. 2

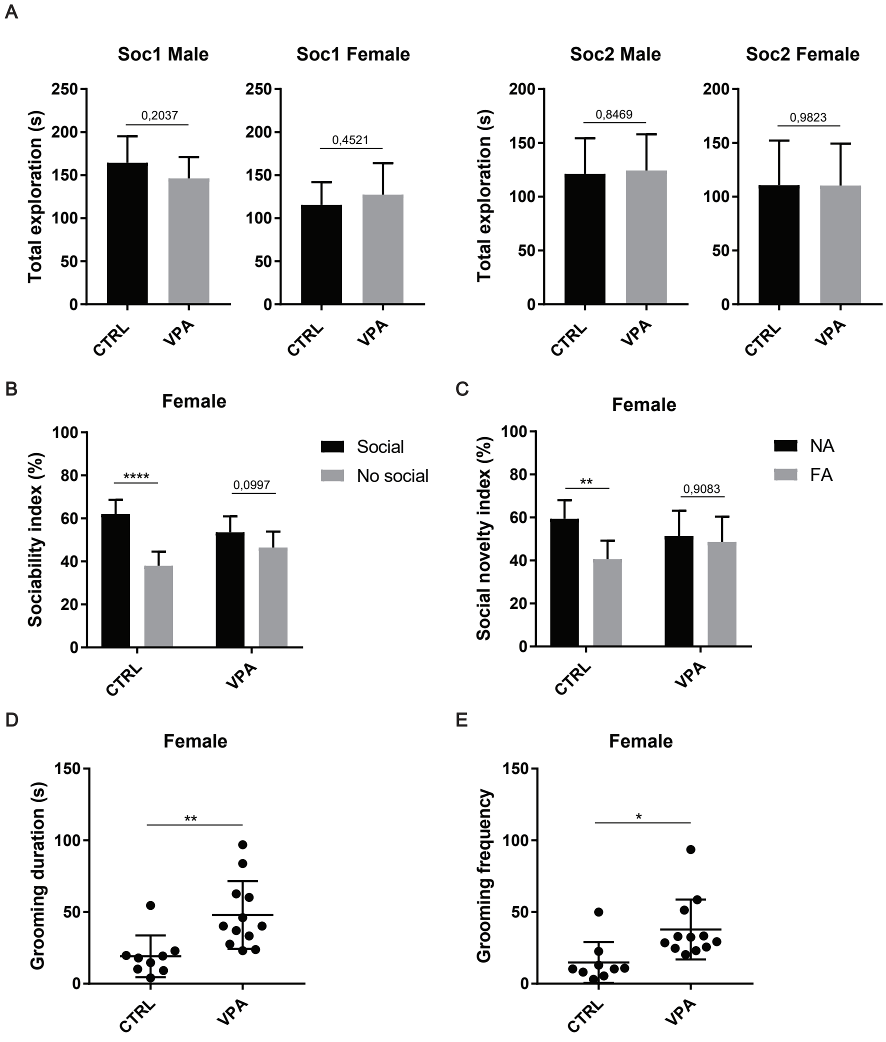
A
Soc1 Male
Soc1 Female
Soc2 Male
Soc2 Female
Total exploration (s)
0,2037
0,4521
0,8469
0,9823
CTRL
VPA
B
Female
Sociability index (%)
Social
No social
****
0,0997
C
Female
Social novelty index (%)
NA
FA
**
0,9083
D
Female
Grooming duration (s)
**
E
Female
Grooming frequency
*

Suppl. 3

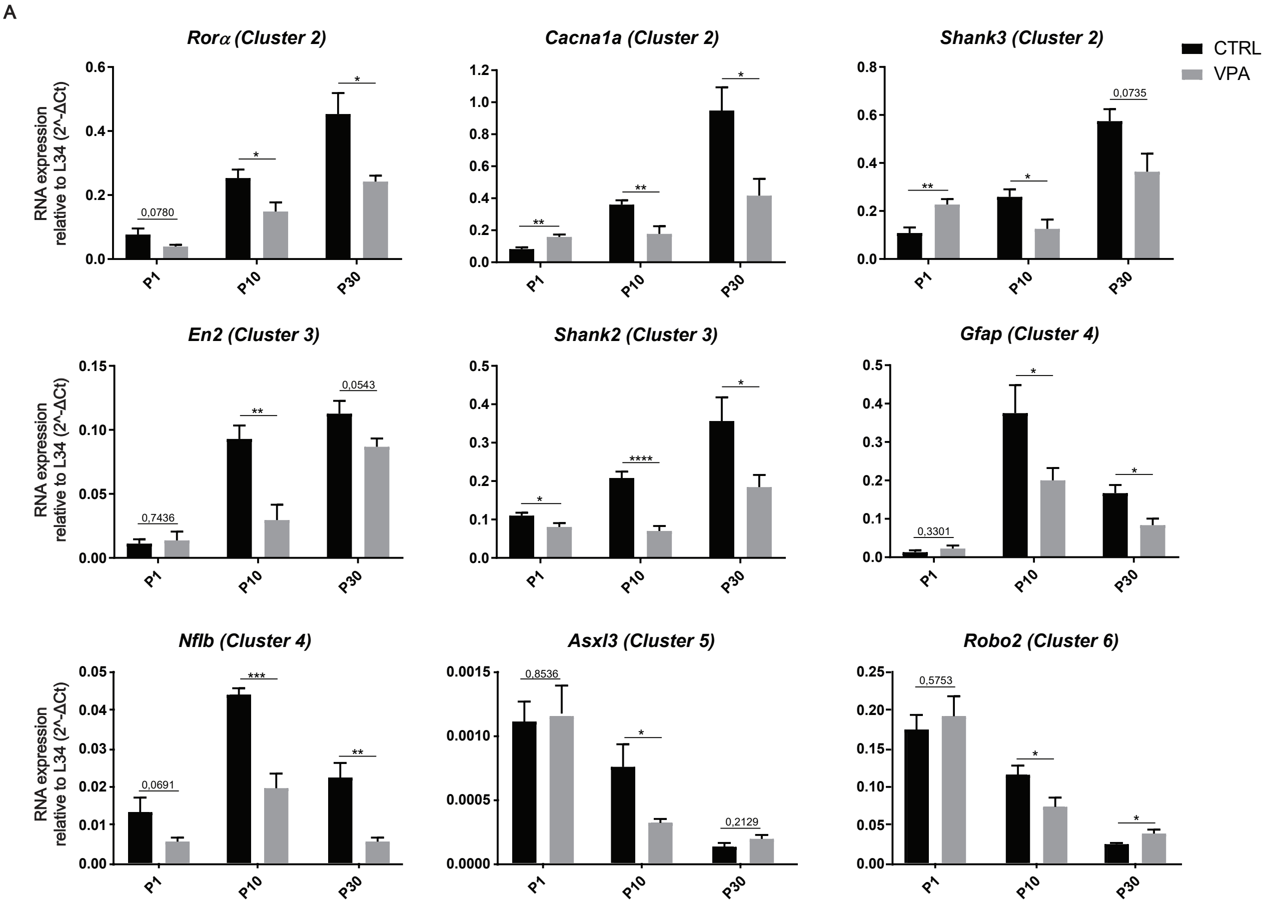
A
Rorα (Cluster 2)
Cacna1a (Cluster 2)
Shank3 (Cluster 2)
CTRL
VPA
En2 (Cluster 3)
Shank2 (Cluster 3)
Gfap (Cluster 4)
Nflb (Cluster 4)
Asxl3 (Cluster 5)
Robo2 (Cluster 6)
RNA expression relative to L34 (2^-ΔCt)
P1
P10
P30
0,0780
0,0735
0,7436
0,0543
0,3301
0,0691
0,8536
0,2129
0,5753
